# Supplementary material for: ADAMTS2 promotes radial migration by activating TGF-β signaling in the developing neocortex
Source: EMBO Rep. 2024 Jun 13;25(7):16. doi: 10.1038/s44319-024-00174-x (PMC11239934; doi:10.1038/s44319-024-00174-x)
Supplement: Supplementary file 1 — Appendix [file 44319_2024_174_MOESM1_ESM.pdf]

APPENDIX

Table of Contents

1. Appendix Figure S1 page 2
2. Appendix Figure S2 page 3
3. Appendix Figure S3 page 4
4. Appendix Figure S4 page 5
5. Appendix Figure S5 page 6
6. Appendix Figure S6 page 7
7. Appendix Figure S7 page 8
8. Appendix Figure S8 page 9
9. Appendix Figure S9 page 10

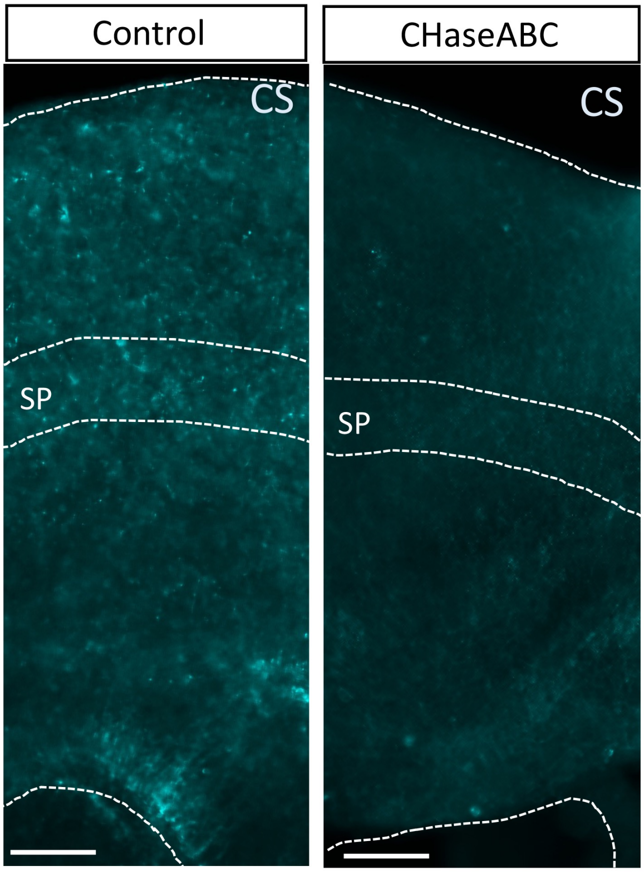

Appendix Figure S1 . The subplate layer is rich in ECM components.
CHase ABC-treated sections used for slice cultures (Fig. 1C and Movie 1) were stained with anti-Chondroitin Sulfate(CS) monoclonal antibody CS-56. CS-immunoreactivities reduced in the treated sections, indicating that CS was degraded. Scale bars;100µm.

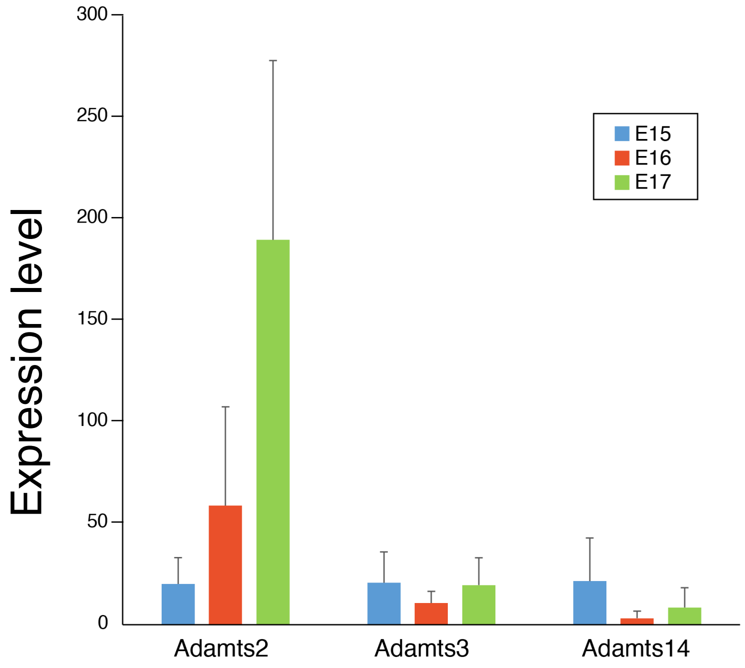

Appendix Figure S2. Changes in expression levels of Adamts 2 subfamily genes expressions during radial neuronal migration

A comparison of the expression levels of ADAMTS2, 3 and 14 subfamilies in FACS-sorted migrating Neurons (N=3: data represents the average values from three independent experiments) .In particular, the expression level of ADAMTS2 was found to be significantlyupregulated during migration.

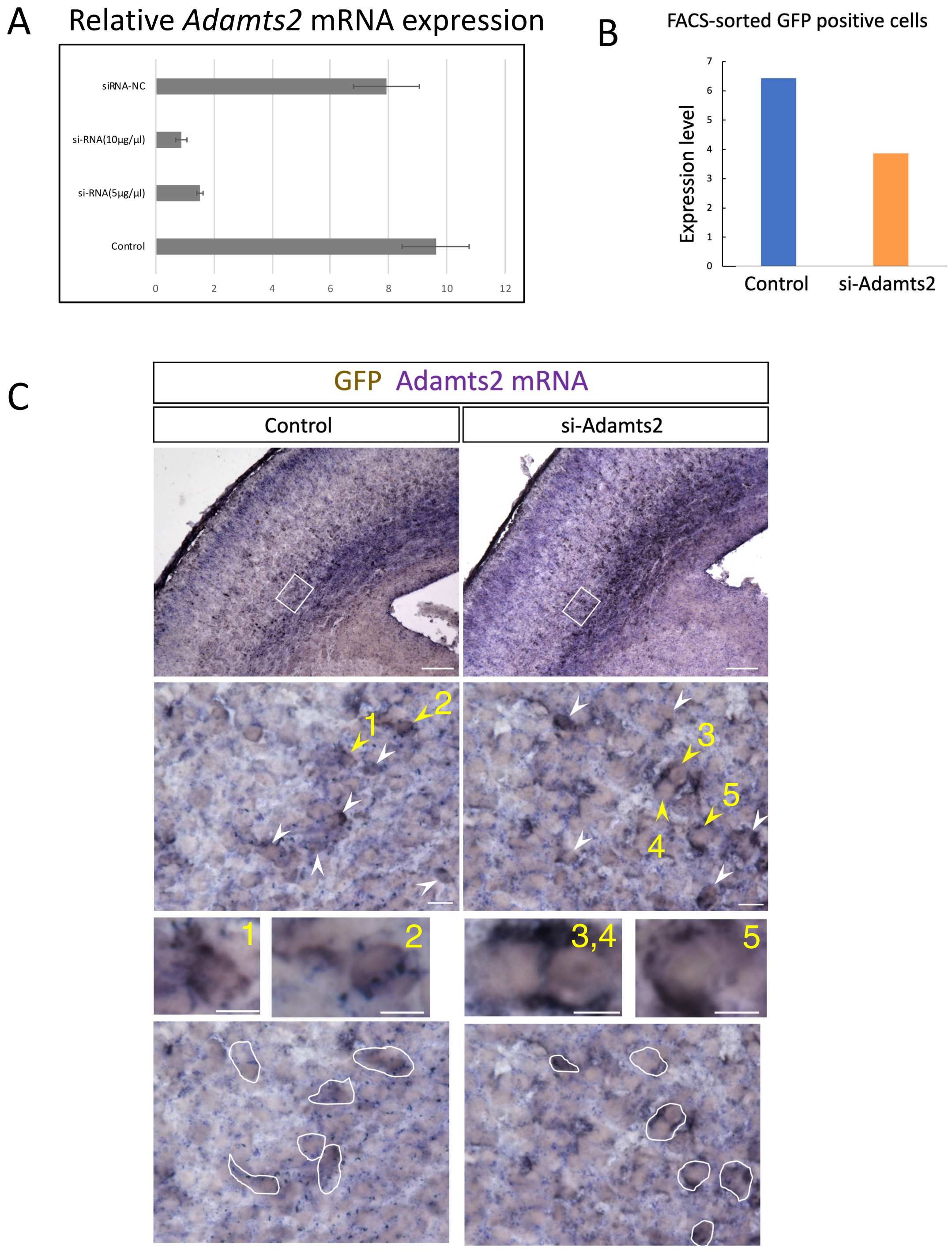

Appendix Figure S3. Verification of Adamts2 knockdown efficiency
A. Efficiency of si-RNA knockdown and the optimal concentration were examined using cultured NIH3T3 cells(N=3 independent experiments). **B.** The si-RNA was electroporated into the fetal mouse brain at E14, then dissection at E17, and GFP-positive cells were isolated by FACS and Q-PCR was performed. Adamts2 expressions were reduced in knockdown samples. **C.** We Confirm the knockdown efficiency by double staining with *in situ* hybridization(ISH) and immunostaining with GFP. Frozen sections of si RNA-electroporated brains were stained with GFP antibody after Adamts2 ISH. Adamts2 mRNA signal (purple) was reduced in GFP-positive si-RNA-transfected knockdown cells (arrowheads) compared to cells in control brains. The enlargement of five cells indicated by yellow arrowheads is shown. The black border-like signal is the GFP antibody signal. The purple dot-shaped signal is the Adamts2 mRNA signal. Scale bars are 50 μm for the lower magnification photographs and 10 μm for the others.

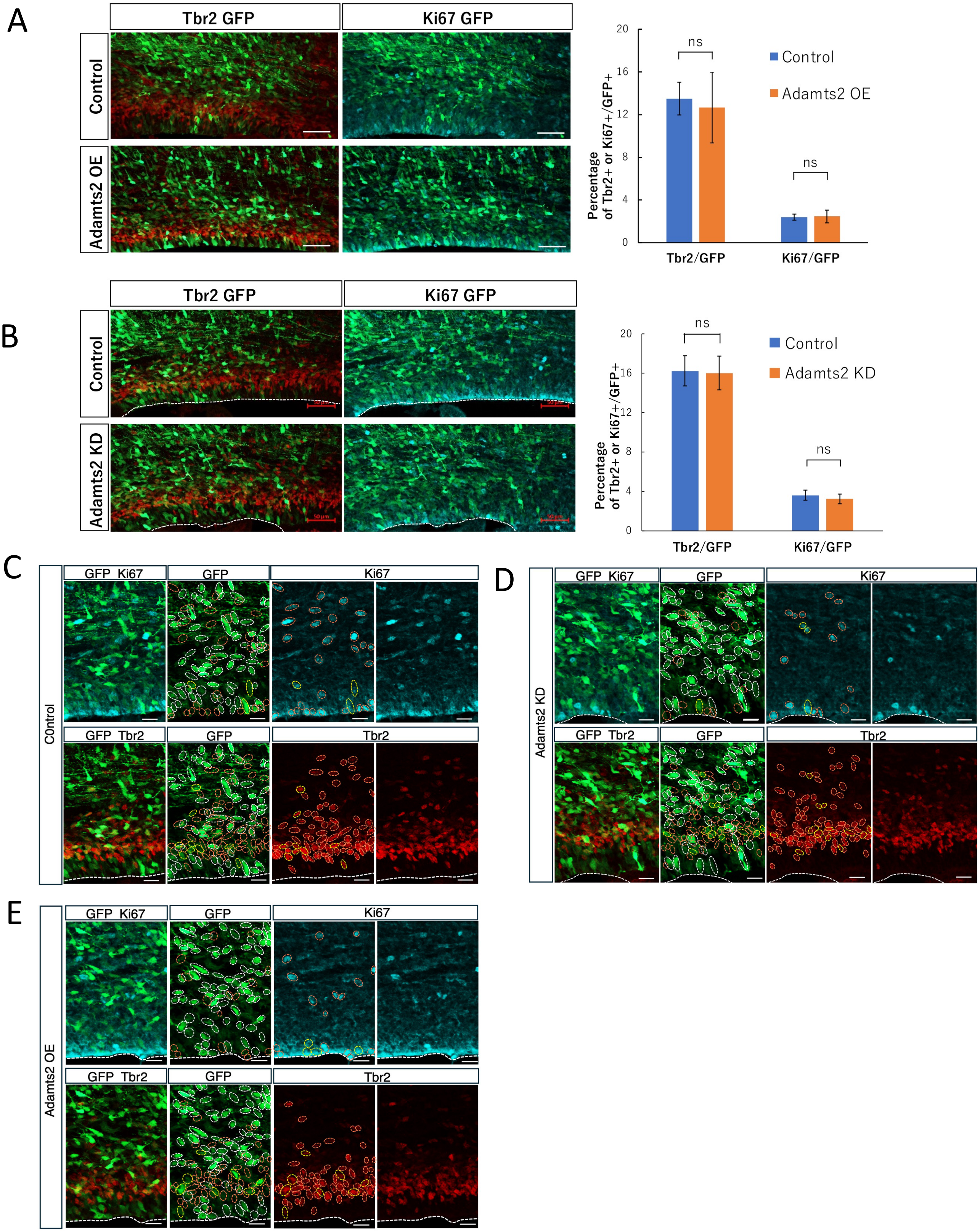

Appendix Figure S4. Overexpression and knockdown of Adamts2 did not alter the neural progenitor cell population. The pND1- Adamts2-expression plasmid (A) or Adamts2 si-RNA (B) along with GFP-expression plasmid was electroporated into the fetal mouse brain at E14. The brains were dissected at E17, and the brain sections were immunostained with anti-Tbr2 (red) or anti-Ki67 (cyan) to label intermediate progenitor cells or mitotic cells, respectively. There were no significant differences in the proportions of Tbr2-positive and Ki67-positive cells between control and the treated samples.(N=4 sections from two brains) (C-E)Example of an actual counted image. GFP, Ki67 and Tbr2 positive cells were each marked, and the number of GFP and double-positive cells were counted to calculate the percentage. (C)Control, (D)Knockdown,(E)Overexpression of Adamts2. Cells circled by white dashed lines are positive for GFP only, yellow lines are GFP positive and double positive for Ki67 or Tbr2, and orange dashed lines are cells positive for Ki67 or Tbr2 alone.Scale bars; 50µm for A,B, 20µm for C,D,E.

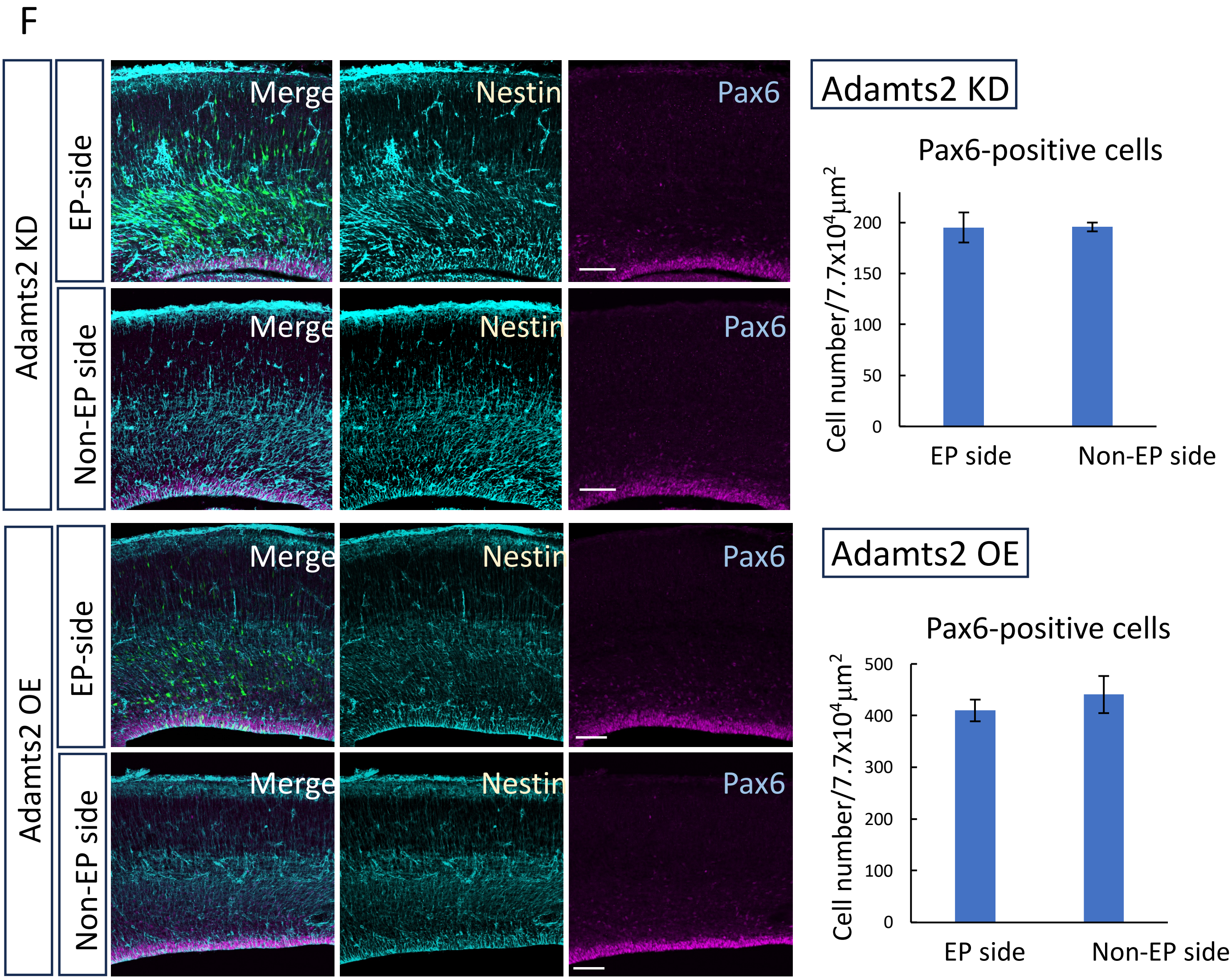

Appendix Figure S5. Overexpression and knockdown of Adamts2 did not alter the integrity of the radial scaffold. Nestin and Pax6 immunostaining was performed to examine the integrity of the radial glial scaffold by comparing the EP-side and non-EP-side of the cortices. The appearance of RG fibers looked the same. The number of all Pax6-positive cells in the area($7.7x10^4mm^2$) of the image was counted, and there was also no significant difference. N=3 sections from three brains. Scale bars; 50μm.

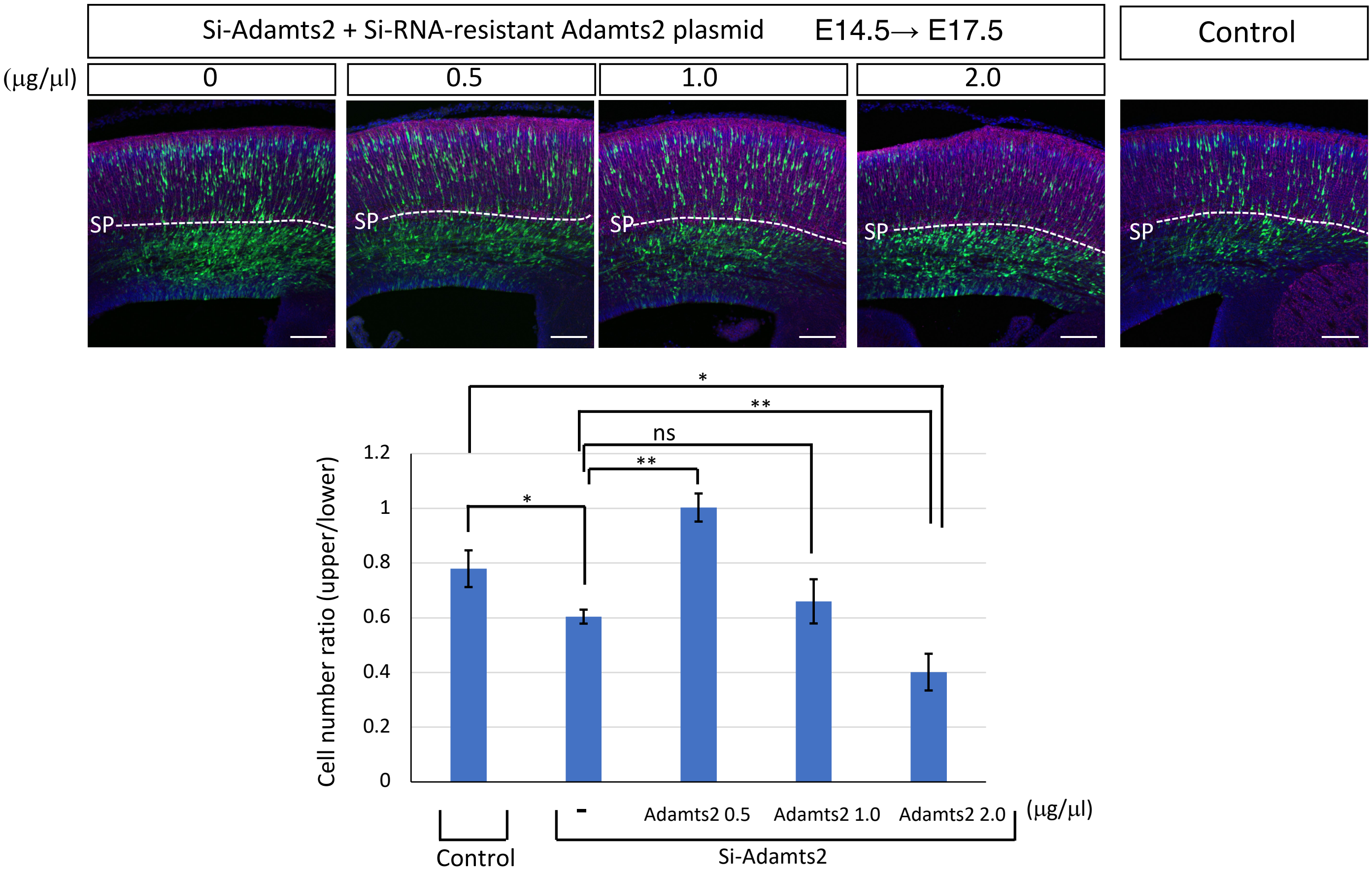

Appendix Figure S6. Overexpression of Adamts2 in the Adamts2 knockdown neurons.
Various concentrations (0.5 µg/µl, 1.0µg/µl and 2.0µg/µl) of si-RNA-resistant Adamts2 expression plasmid was introduced into neural progenitors along with si-RNA for Adamts2 by *in utero* electroporation at E14.5, and the brains were dissected at E17.5. At 0.5 µg/µl, the most efficient rescue was observed. At 2.0µg/µl, the migration was rather suppressed. N=5 sections from two brains each, derived from two mother mice. Scale bars; 100µm.

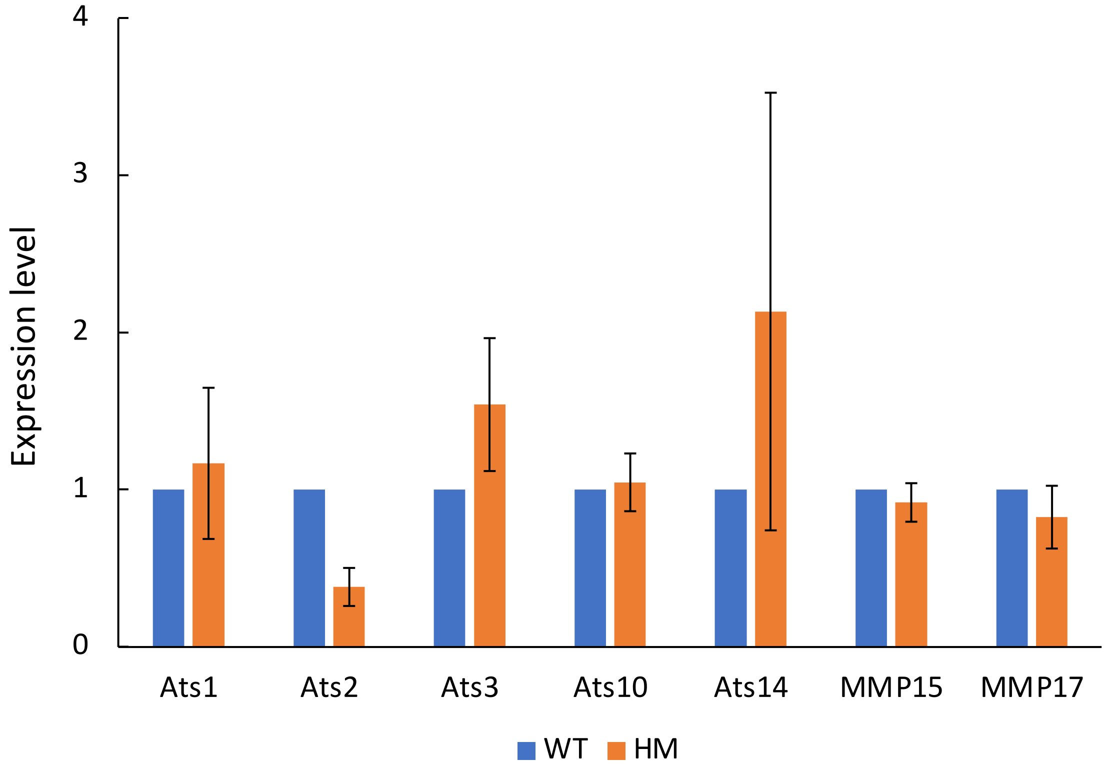

Appendix Figure S7. Investigation of compensatory expression of other ECM proteases in Adamts2KO homozygous cortex. RNA was extracted from the fetal brain (E17)of Adamts2 KO mice (homozygous and WT), and Q -PCR was performed. Results represent the expression of homozygous when the WT expression level is set to 1. A strend towards Adamts3 and 14 being upregulated in homozygotes were seen, although the difference was not statistically significant. N=3 brains each.

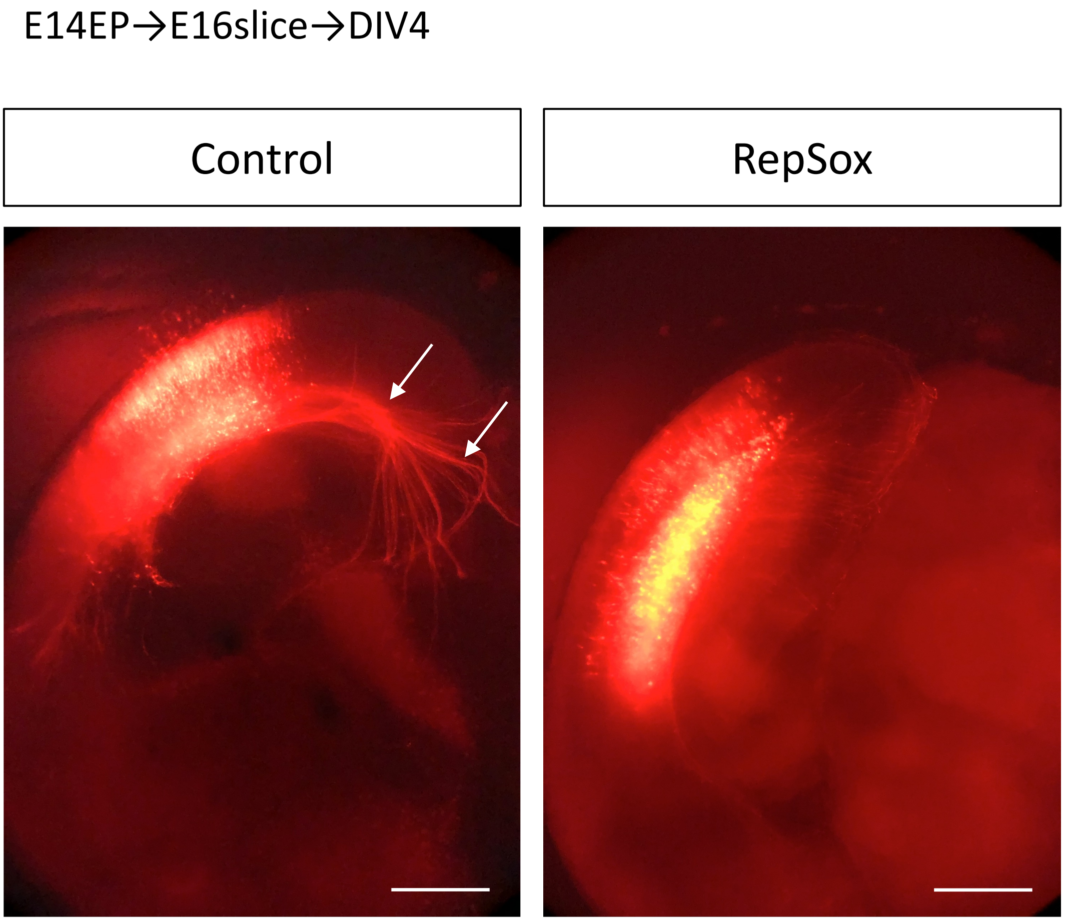

Appendix Figure S8. TGFβR inhibitor impaired migration and inhibited axon elongation. RFP expression plasmids were introduced into neural progenitor cells by *in utero* electroporation at E14, and the brain slices were prepared at E16. After time-lapse imaging, slices were fixed at DIV4. In the control slices, neuronal migration and extension of axon tracts (arrows) were observed, both of which were suppressed in the RepSox-treated slices. Scale bars; 200µm

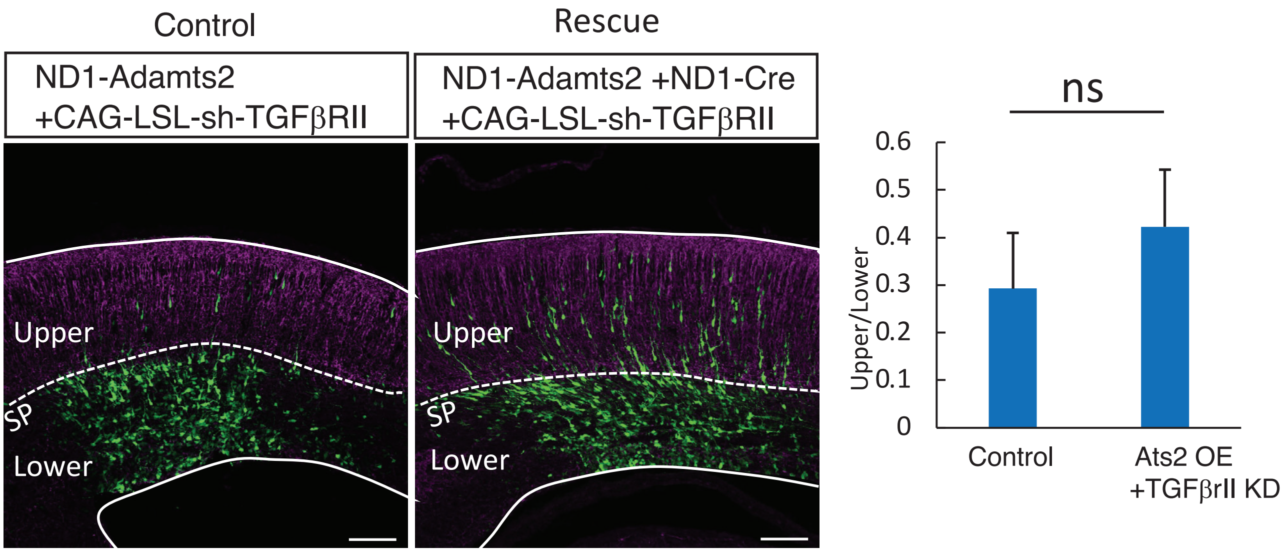

Appendix Figure S9. Migratory defects in overexpression of Adamts2 in neurons tend to be rescued by the knockdown of TGFβRII in neurons. Both overexpression of Adamts2 and shRNA knockdown of TGFβRII were performed under the NeuroD1 promoter. NeuroD1-Adamts2 and NeuroD1-LSL-shTGFβRII plasmids were electroporated in utero together with(Rescue) or without(Control) NeuroD1-Cre at E14 and the brains were dissected at E17. N=4 sections from four brains collected from two mother mice. The statistical significance for each pair of the same bin was measured by unpaired, two-tailed t-tests (*p $<$ 0.05; **p $<$ 0.01; ***p $<$ 0.001, ns; not significant).
