# Supplementary material for: ADAMTS2 promotes radial migration by activating TGF-β signaling in the developing neocortex
Source: EMBO Rep. 2024 Jun 13;25(7):16. doi: 10.1038/s44319-024-00174-x (PMC11239934; doi:10.1038/s44319-024-00174-x)
Supplement: Supplementary file 6 — Movie EV3 [file 44319_2024_174_MOESM6_ESM.zip › Movie EV3.docx]

Movie EV3

**Inhibitors of TGF-β receptor (50 μM RepSox) affected radial migration in cultured slices**

CAG-Lifeact (F-actin labelling) and RFP plasmids were electroporated at E14.5, and cultured slices were prepared at E16.5. Time-lapse imaging was performed for 11 h in the presence or absence of 50 μM RepSox. The multipolar-to-bipolar transition was impaired in the presence of the inhibitor. Arrows indicate neurites. In the control, cells became bipolar and had a leading process, while those in the TGF-β inhibitor treated group were observed to have multipolar neurites.
