# Supplementary material for: ADAMTS2 promotes radial migration by activating TGF-β signaling in the developing neocortex
Source: EMBO Rep. 2024 Jun 13;25(7):16. doi: 10.1038/s44319-024-00174-x (PMC11239934; doi:10.1038/s44319-024-00174-x)
Supplement: Supplementary file 7 — Movie EV4 [file 44319_2024_174_MOESM7_ESM.zip › Movie EV4.docx]

Movie EV4

**Visualization of TGF-β signaling during radial migration with the luminescence imaging probe**

In the control, the luminescence signals were detected in the lower part of the SP layer and in the upper part of the intermediate zone (top right panel). The top left panel shows a movie of a GFP-detecting channel. The luminescence signal was specifically suppressed in the presence of RepSox (bottom right panel), confirming that this signal is indeed a **TGF-β** signaling. Luminescence signals were significantly reduced in Adamts2 knockdown neurons (bottom left panel), indicating that ADAMTS2 is required to activate TGF-β signaling.
