# Supplementary material for: ADAMTS2 promotes radial migration by activating TGF-β signaling in the developing neocortex
Source: EMBO Rep. 2024 Jun 13;25(7):16. doi: 10.1038/s44319-024-00174-x (PMC11239934; doi:10.1038/s44319-024-00174-x)
Supplement: Supplementary file 8 — Expanded View Figures [file 44319_2024_174_MOESM8_ESM.pdf]

Expanded View Figures

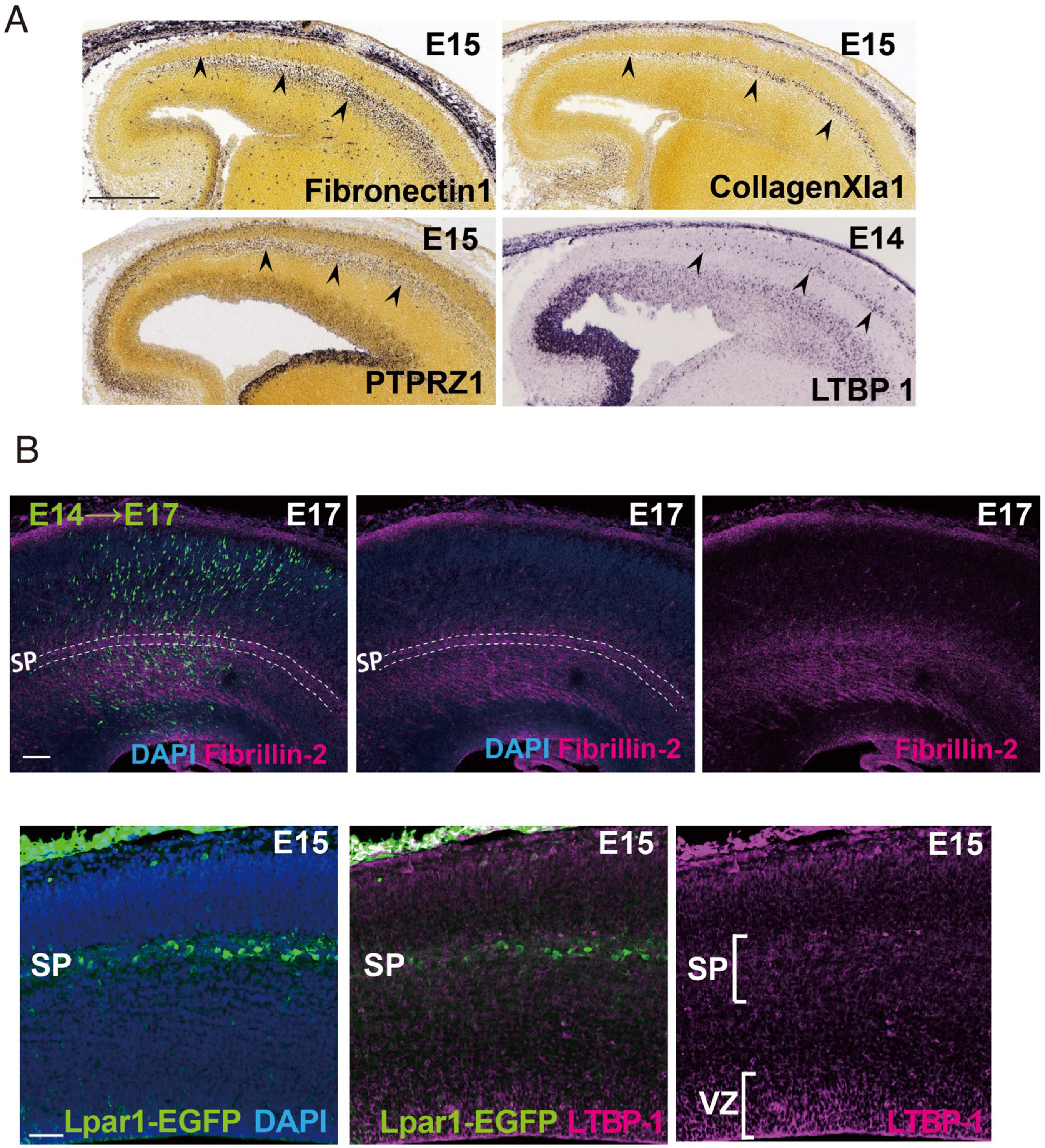

Figure EV1. The subplate layer is rich in ECM components.

(**A**) In situ hybridization databases revealed that mRNAs of genes encoding ECM proteins are localized at the subplate layer in the developing mouse cortex (arrowheads). The data for Fibronectin 1, Collagen XIa1, and PTPRZ1 are from Allen brain atlas and the data for LTBP1 is from Gene Paint. (**B**) Immunohistochemistry of Fibrillin-2 indicated that this protein is localized at subplate (SP) and intermediate zone. The migrating neurons were labeled with EGFP by in utero electroporation at E14. The immunoreactivities for LTBP-1 were localized at SP and ventricular zone (VZ). In this section, SpNs were labeled by EGFP.

© The Author(s)

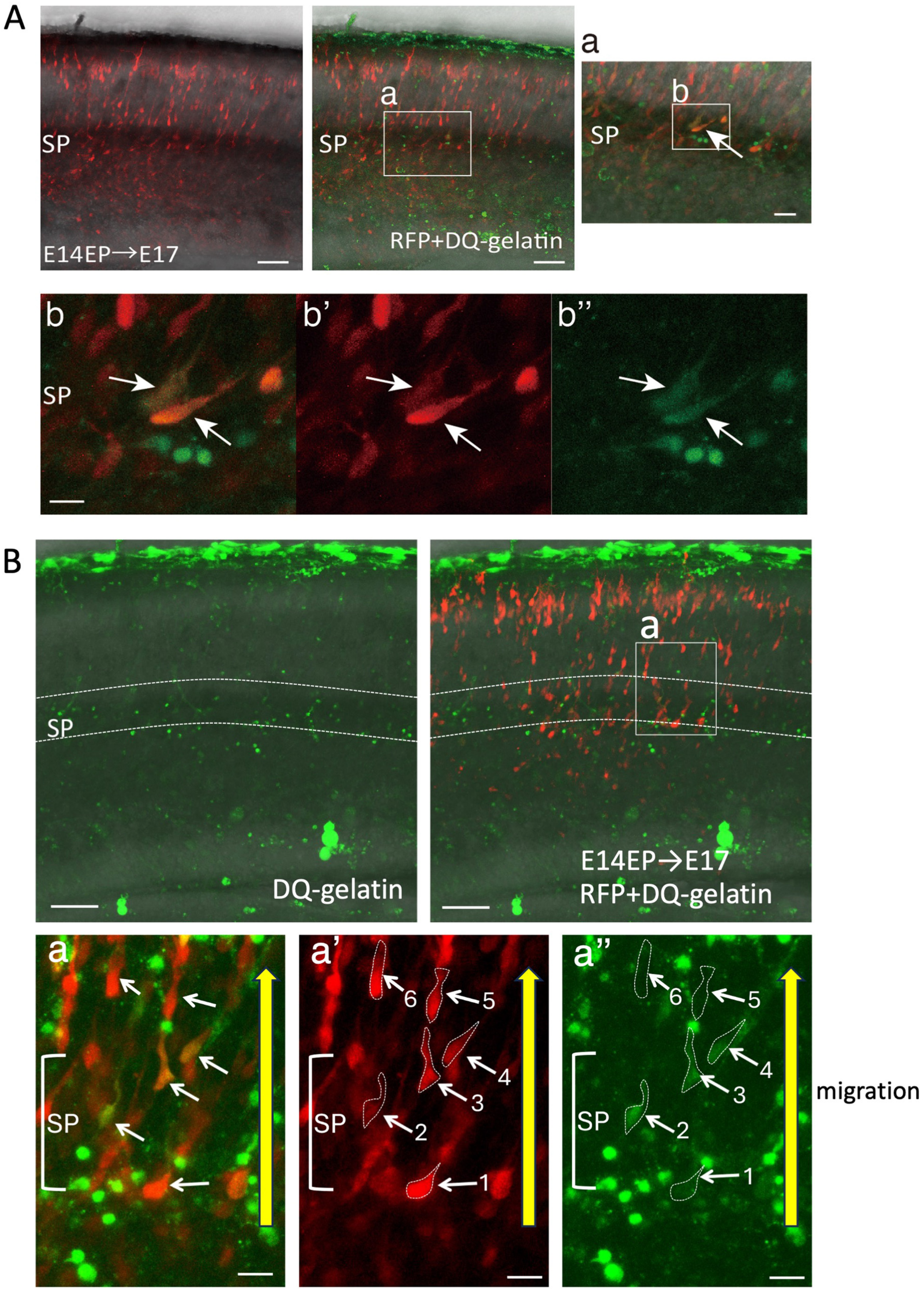
A
SP
E14EP→E17
a
SP
RFP+DQ-gelatin
a
b
SP
b
SP
b'
b''
B
SP
DQ-gelatin
a
E14EP→E17
RFP+DQ-gelatin
a
SP
a'
6
5
4
3
SP
2
1
a''
6
5
4
3
SP
2
1
migration

 © The Author(s)

Figure EV2. In situ zymography using DQ-gelatin revealed that ECM protease activity (green) occurred in the SP layer.

(**A**) RFP expression plasmids were electroporated in utero at E14.5, and brains were dissected at E17.5. Cultured slices were prepared using these electroporated brains and were incubated with DQ-gelatin. Image acquisition began 30 min after incubation, and time-lapse imaging was performed every 10 min for ~16 h. Enlarged images reveal that the migrating neurons exhibited gelatinolytic activities near the SP layer (indicated by arrows in a–b″). (**B**) Example of another slice. When the region (a) is enlarged, cells before entering the SP (cell 1) are green-negative, but cells entering the SP (cells 2, 3 and 4) are green-positive and show gelatinase activity. However, cells that have passed through the SP layer (cells 5, 6) are also green-negative. Scale bars, 50 μm for (**A**), (**B**), 10 μm for (**A**-a-b″) and (**B**-a-a″).

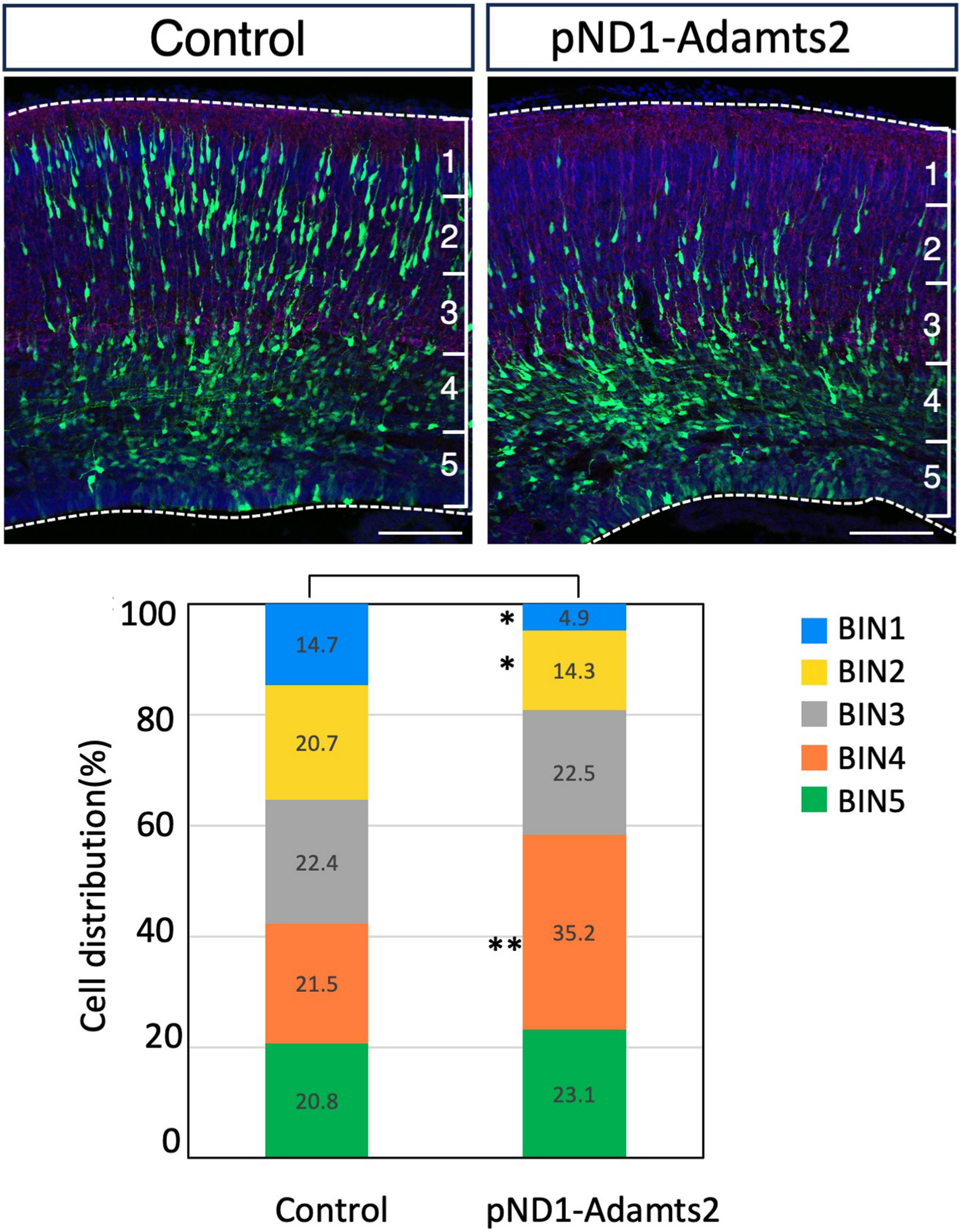
Control
pND1-Adamts2
1
2
3
4
5
100
80
60
40
20
0
Cell distribution(%)
14.7
20.7
22.4
21.5
20.8
*
4.9
*
14.3
22.5
**
35.2
23.1
BIN1
BIN2
BIN3
BIN4
BIN5
Control
pND1-Adamts2

© The Author(s)

Figure EV3. Neuron-specific overexpression of Adamts2.

Adamts2 cDNA was subcloned under the NeuroD1 promoter and used for in utero electroporation experiments. In utero electroporation was performed at E14, and the brains were dissected at E17. The number of cells distributed in the five bins was counted. Neuron-specific overexpression of Adamts2 resulted in impaired migration ($N = 5$ sections for each group; two fetuses from two mother mice were collected, and we used one or two sections from each brain for quantification. Control and overexpression were counted in pairs on the same litter) The statistical significance for each pair of the same bin was measured by unpaired, two-tailed t-tests (*$p < 0.05$; **$p < 0.01$; ***$p < 0.001$) Scale bars; 100 μm.

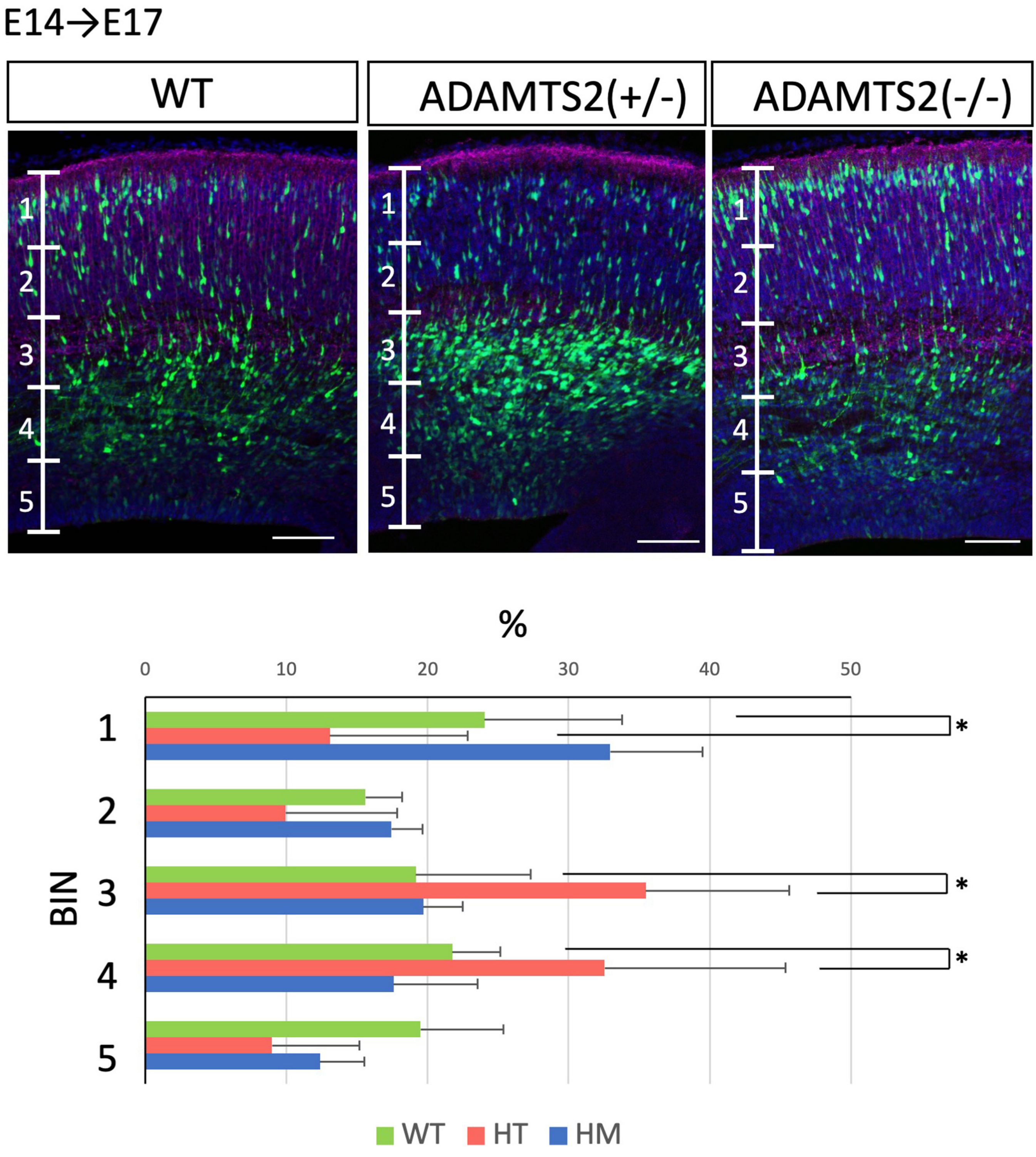

Figure EV4. Impaired radial neuronal migration in the brain of Adamts2 knockout heterozygous mice.

Adamts2 KO mice showed significant migration defects in heterozygous (HT) mice, but not in homozygous (HM) mice. $N = 6$ sections; two fetuses from three mother mice for WT and HT, $N = 4$ sections; two fetuses from two mother mice for HM. The statistical significance for each pair of the same bin was measured by unpaired, two-tailed t-tests (*$p < 0.05$; **$p < 0.01$; ***$p < 0.001$). Scale bars; 100 μm.

 © The Author(s)

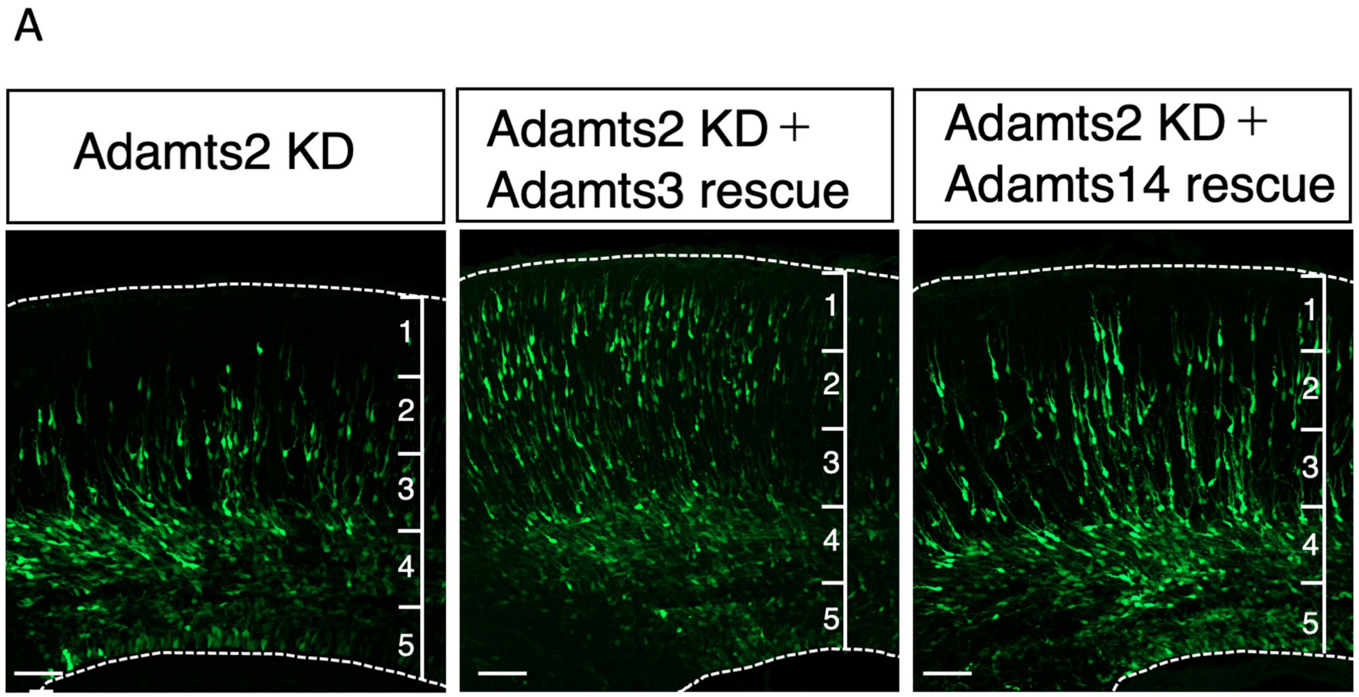
A
Adamts2 KD
Adamts2 KD + Adamts3 rescue
Adamts2 KD + Adamts14 rescue
1
2
3
4
5

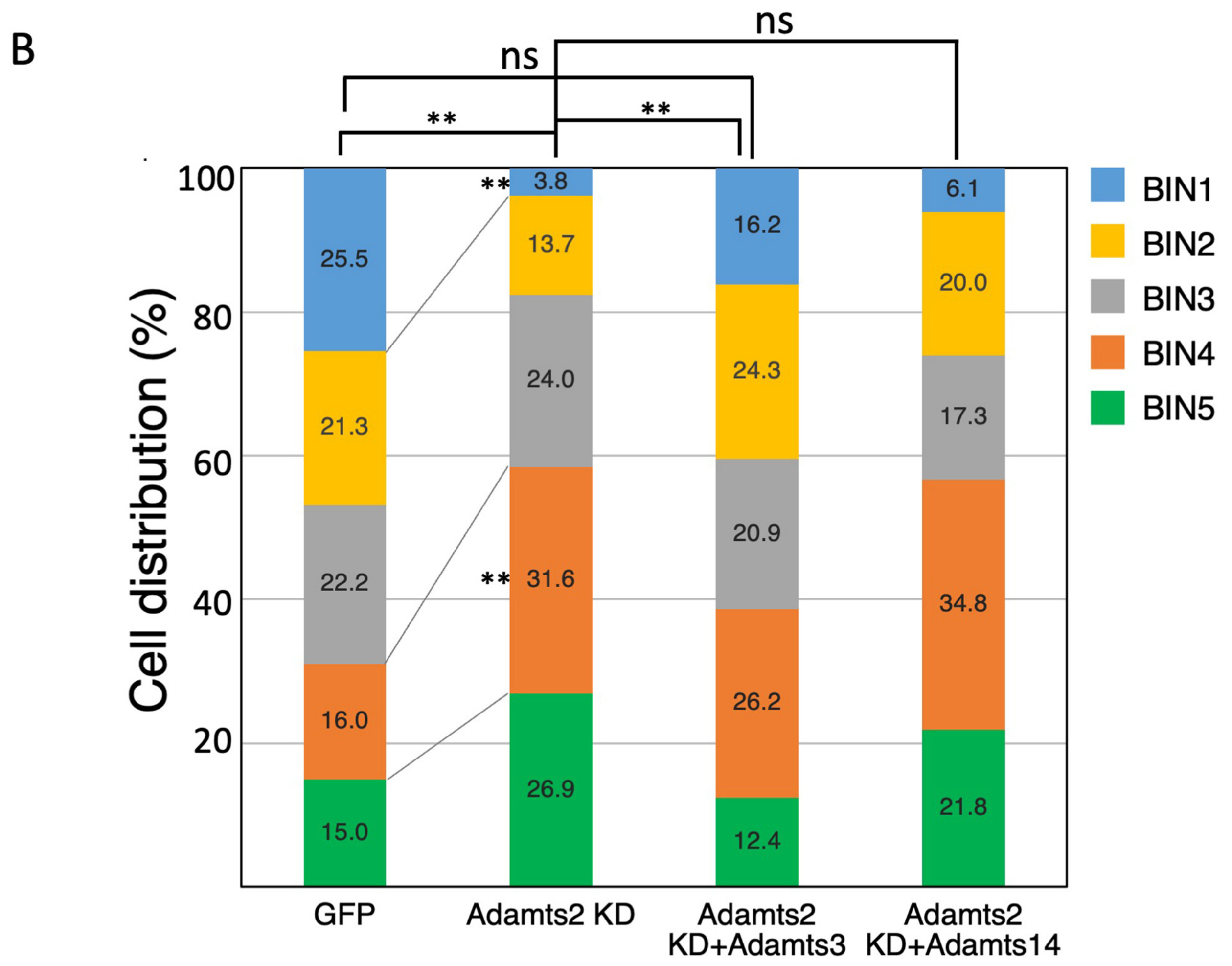
B
ns
ns
**
**
Cell distribution (%)
100
80
60
40
20
25.5
21.3
22.2
16.0
15.0
** 3.8
13.7
24.0
** 31.6
26.9
16.2
24.3
20.9
26.2
12.4
6.1
20.0
17.3
34.8
21.8
BIN1
BIN2
BIN3
BIN4
BIN5
GFP
Adamts2 KD
Adamts2 KD+Adamts3
Adamts2 KD+Adamts14

Figure EV5. Adamts3 rescued the migration defects caused by Adamts2 knockdown.

Adamts3 and Adamts14 cDNAs were subcloned directly under the NeuroD1 promoter and used for the rescue experiments of Adamts2 knockdown. In utero electroporation was performed at E14, and the brains were dissected at E17 (**A**). The number of cells distributed in the five bins was counted (**B**). When Adamts3-expression plasmid was introduced with Adamts2 si-RNA, the migration phenotype was rescued. In the case of Adamts14, the migration phenotype was not rescued. $N = 5$–7 slices from three brains to six brains collected from two mother mice were used for the analysis. The statistical significance for each pair of the same bin was measured by unpaired, two-tailed t-tests (*$p < 0.05$; **$p < 0.01$; ***$p < 0.001$). Scale bars, 50 mm.

 © The Author(s)
